# Segmentation and Multi-Timepoint Tracking of 3D Cancer Organoids from Optical Coherence Tomography Images Using Deep Neural Networks

**DOI:** 10.3390/diagnostics14121217

**Published:** 2024-06-08

**Authors:** Francesco Branciforti, Massimo Salvi, Filippo D’Agostino, Francesco Marzola, Sara Cornacchia, Maria Olimpia De Titta, Girolamo Mastronuzzi, Isotta Meloni, Miriam Moschetta, Niccolò Porciani, Fabrizio Sciscenti, Alessandro Spertini, Andrea Spilla, Ilenia Zagaria, Abigail J. Deloria, Shiyu Deng, Richard Haindl, Gergely Szakacs, Agnes Csiszar, Mengyang Liu, Wolfgang Drexler, Filippo Molinari, Kristen M. Meiburger

**Affiliations:** 1 Biolab, Polito^BIO^Med Lab, Department of Electronics and Telecommunications, Politecnico di Torino, Corso Duca degli Abruzzi 24, 10129 Turin, Italy; francesco.branciforti@polito.it (F.B.); massimo.salvi@polito.it (M.S.); s303868@studenti.polito.it (F.D.); francesco.marzola@polito.it (F.M.); s294796@studenti.polito.it (S.C.); s289534@studenti.polito.it (M.O.D.T.); s297655@studenti.polito.it (G.M.); s295351@studenti.polito.it (I.M.); s297328@studenti.polito.it (M.M.); s296287@studenti.polito.it (N.P.); fabrizio.sciscenti@polito.it (F.S.); s305486@studenti.polito.it (A.S.); s305286@studenti.polito.it (A.S.); s298537@studenti.polito.it (I.Z.); filippo.molinari@polito.it (F.M.); 2 Center for Medical Physics and Biomedical Engineering, Medical University of Vienna, 1090 Vienna, Austria; abigail.deloria@meduniwien.ac.at (A.J.D.); shiyu.deng@meduniwien.ac.at (S.D.); richard.haindl@meduniwien.ac.at (R.H.); mengyang@udel.edu (M.L.); wolfgang.drexler@meduniwien.ac.at (W.D.); 3 Center for Cancer Research, Medical University of Vienna, 1090 Vienna, Austria; gergely.szakacs@meduniwien.ac.at (G.S.); agnes.csiszar@meduniwien.ac.at (A.C.)

**Keywords:** organoids, cancer, deep learning, optical coherence tomography, segmentation, tracking

## Abstract

Recent years have ushered in a transformative era in in vitro modeling with the advent of organoids, three-dimensional structures derived from stem cells or patient tumor cells. Still, fully harnessing the potential of organoids requires advanced imaging technologies and analytical tools to quantitatively monitor organoid growth. Optical coherence tomography (OCT) is a promising imaging modality for organoid analysis due to its high-resolution, label-free, non-destructive, and real-time 3D imaging capabilities, but accurately identifying and quantifying organoids in OCT images remain challenging due to various factors. Here, we propose an automatic deep learning-based pipeline with convolutional neural networks that synergistically includes optimized preprocessing steps, the implementation of a state-of-the-art deep learning model, and ad-hoc postprocessing methods, showcasing good generalizability and tracking capabilities over an extended period of 13 days. The proposed tracking algorithm thoroughly documents organoid evolution, utilizing reference volumes, a dual branch analysis, key attribute evaluation, and probability scoring for match identification. The proposed comprehensive approach enables the accurate tracking of organoid growth and morphological changes over time, advancing organoid analysis and serving as a solid foundation for future studies for drug screening and tumor drug sensitivity detection based on organoids.

## 1. Introduction

Recent advancements have significantly changed the field of in vitro modeling thanks to the progression of research using organoids. Organoids are intricate, three-dimensional structures that autonomously form through self-organization using stem cells or neoplastic cells derived directly from patients and animal models [1,2]. Organoids also exhibit greater similarities to derived organs in terms of both morphological and functional characteristics [3] which make them essential tools for developmental biology and disease studies. Non-destructive analysis over long time periods is an unmet need of the organoid research field, as some organoid models and assays require weeks to months to be established [4,5]. Current gold standard methods either necessitate endpoint analysis, manipulation with fluorescent dyes, or are 2D imaging methods that only give a rough estimation of organoid size and barely any on organoid morphology. However, enabling exact organoid tracking over prolonged time allows for a more accurate assessment of the organoid’s characteristics, e.g., differentiation status as reported on the identification of morphological and marker changes, for example, in retinal organoids [6], disease progression in liver, intestinal, and brain organoids [7], and the drug effect in cancer organoids [8]. As cancer organoids have been reported to mimic patients’ drug response [9], the establishment of pipelines for longitudinal monitoring with exact volumetric assessment is of high direct translational value for clinics.

By mimicking tumors’ inter- and intra-heterogeneity in vitro, cancer organoids provide a platform for exploring tumor progression, therapy resistance, and precise drug screening [10,11]. Realizing the full potential of organoids requires advanced imaging technologies to enable a comprehensive exploration of organoid structures and behaviors, as it is of paramount importance to be able to analyze organoid growth in a non-destructive and label-free manner to facilitate discoveries and advancements in personalized medicine [12].

Optical coherence tomography (OCT) is a promising imaging modality for organoid analysis, as it can offer high-resolution, label-free, non-destructive, and real-time 3D imaging of biological tissues [13,14] and vasculature if multiple volumes are acquired at the same location through OCT angiography [15]. The label-free and non-destructive nature of OCT is pivotal as it permits the longitudinal monitoring of organoids, allowing a quantitative analysis of numerous important features of single organoid growth and status, such as volume and internal structures [8,16,17]. In fact, within the dynamic field of organoid imaging, advanced analytical techniques are crucial for understanding complex morphological changes and developmental processes [1]. Machine learning (ML) and deep learning (DL) methods, such as convolutional neural networks (CNNs), are essential for extracting detailed features and tracking developmental stages in organoids. Despite its potential, accurately identifying and quantifying organoids in OCT images remains challenging due to poor contrast, unclear boundaries, noise, and the small size of organoids at the beginning of cultivation when starting at single cells. Numerous solutions have been proposed in the literature for the segmentation and tracking of organoids, but the majority employ imaging modalities such as fluorescence microscopy or traditional microscopy [18,19,20]. In the context of organoid segmentation in OCT images, an initial study proposed by Gil et al. in 2021 [8] demonstrated the feasibility of volumetrically tracking organoid growth in patient-derived cancer organoids. The authors proposed an automated image analysis framework based on preprocessing, registration, segmentation using k-means clustering, and a centroid particle tracking algorithm to track individual organoids over multiple timepoints covering 48 h. Recently, Bao et al. [21] employed deep convolutional neural networks for organoid segmentation and demonstrated the feasibility of detecting and tracking the growth of 3D bio-printed organoid clusters in OCT volumes using a deep learning approach. The authors imaged the organoids over 7 days at an interval of every 24 h, and the results of the study also demonstrated the influence of the organoid scale on segmentation accuracy. The study by Bao et al. importantly demonstrates the reconstruction of organoid clusters’ multiscale structure and the quantitative analysis of growth dynamics using deep learning but shows some important limitations. Firstly, the division of the dataset is not clearly described, which could influence the method’s capability to generalize to new OCT volume acquisitions. Secondly, the method allows for proper tracking over different timepoints but is still partially limited by extension, as organoid growth is followed for 7 days.

To address the current limitations and further show the potential to track volume changes in tumor organoids, we propose an OCT-based deep learning method with convolutional neural networks (CNNs). The main contributions of this paper can be summarized as follows:

- The development of an optimized preprocessing step to highlight organoids within the noisy OCT volume.
- The implementation of a state-of-the-art deep learning model and ad hoc postprocessing methods, showing good generalizability on an external test set.
- The development of an automatic algorithm for the quantitative analysis of organoid growth dynamics, demonstrating results over an extensive 13 days.

## 2. Materials and Methods

**Figure 1:**
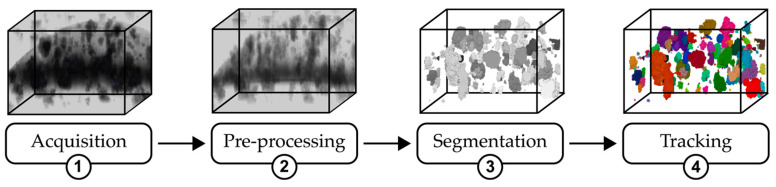
An overview of the proposed pipeline: (1) acquisition, where the raw image volume is captured; (2) preprocessing, illustrating the enhancement techniques applied to improve image quality; (3) segmentation, showing the result of organoid segmentation from the enhanced image; and (4) tracking, depicting the final stage where individual organoids are identified and labeled across sequences.

The implemented pipeline acquires insights into the behavior and morphological characteristics of organoids over time from OCT volumes, as illustrated in Figure 1. Initially, to improve the overall quality of the images, volumes undergo a series of custom preprocessing steps designed to enhance the visibility of organoids and increase the signal-to-noise ratio. Given the high intrinsic variability of these types of images, a CNN is employed to generate binary masks. These masks are then subjected to a postprocessing phase aimed at enhancing their accuracy. Finally, the masks are used to extract critical information for the biological analysis of organoids and for their tracking over time.

### 2.1. Organoid Preparation and Culture and Image Dataset

Murine BRCA1-deficient breast cancer organoids oKB1P4s were used and cultivated as described [22]. For the OCT longitudinal experiment, 1000 cells were seeded at a 50:50 ratio of media and basal membrane extract (Cultrex BME, bio-techne R&D Systems, Minneapolis, MN, USA) in 8-well plates (Ibidi, Gräfelfing, Germany), incubated for 45 min at 37 °C, then covered with 300 µL media and cultivated at 37 °C, 5% CO_2_. On the third day, the culture was subjected to 8 µM carboplatin for 5 days, after which normal media were used for replacements.

Organoid imaging was performed using a home-built spectral-domain OCT system using a laser source (EBD290002, EXALOS, Schlieren, Switzerland) with 845 nm central wavelength and 131 nm bandwidth [23]. The OCT system has a lateral resolution of 3.4 µm and an axial resolution of 2.68 µm in tissue (a refractive index of 1.38 was taken). A 3.0 mm × 1.8 mm field-of-view was scanned with a step size of 2.52 µm in the transverse directions at a scan speed of 10 kHz. After acquisition, the organoid data underwent the standard OCT processing procedure, including background removal, resampling, and fast Fourier transform, as well as acquisition artifact correction.

**Figure 2:**
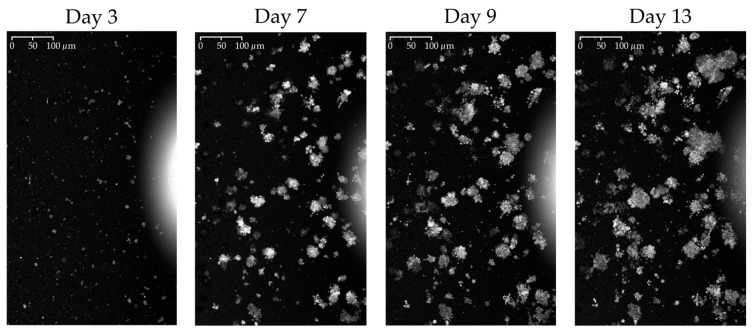
MIPs from the top view of the same organoid culture at different timepoints. The sequential images highlight the dynamic growth of the organoids, capturing the progressive changes in size and structure through various stages of development.

The acquired OCT volumes were then systematically partitioned into training, validation, and testing sets to facilitate the development and assessment of the proposed algorithm. Volumes from samples w1 and w2, each comprising 5 timepoints, were allocated for algorithm training and validation. In contrast, sample w3, which includes a broader temporal range with 7 timepoints, was reserved for testing the algorithm across a more diverse set of time steps. Each volume consists of 698 slices with dimensions of 1200 × 800 pixels. Table 1 provides a comprehensive overview of the data utilized in this study, while Figure 2 shows an example of the maximum intensity projection (MIP) images of unprocessed OCT volumes.

### 2.2. Preprocessing

**Figure 3:**
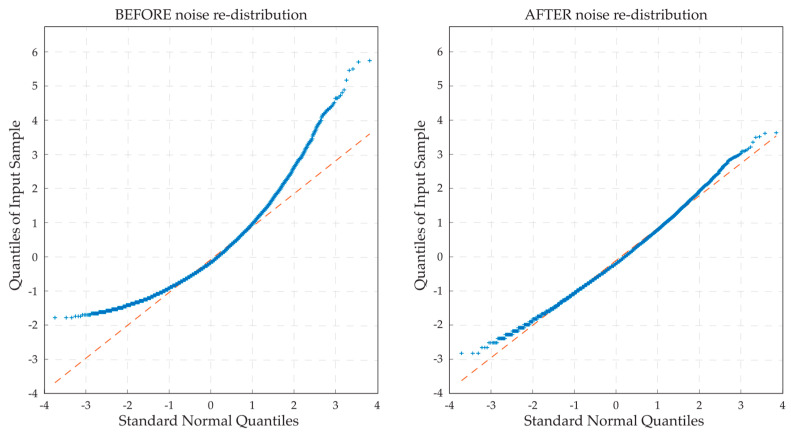
Quantile–quantile (QQ) plots showing noise intensity distribution before (**left**) and after (**right**) the square root transformation, from a sample volume excluding the organoids and regions outside the drop. In these plots, the *x*-axis represents the theoretical quantiles of a standard normal distribution, and the *y*-axis represents the observed quantiles of noise intensities. A closer alignment to the red dashed reference line indicates that the noise distribution conforms more closely to a Gaussian distribution.

The dataset consists of OCT scans stored in 16-bit unsigned integer (uint16) format. The preprocessing aims to standardize intensity, enhance contrast, and reduce noise. First, histogram normalization is applied based on the 95th percentile of the summed histograms from the training set volumes [24]. This standardizes intensity dynamics across scans. The normalized volume $V^{\prime }$ is obtained as follows:

(1) $$V^{\prime }\;=\;\left(\frac{p_{95}^{S}}{p_{95}}\right)\cdot V$$

where V is the original image data, and $p_{95}$ is the 95th percentile from its luminance histogram H. $p_{95}^{S}$ is the 95th percentile from the reference histogram $H_{S}$, which is defined as follows:

(2) $$H_{S}=\sum_{j\in T}H_{j}$$

where T represents the set of volumes belonging to the training set. Next, histogram stretching is conducted between the 1st and 99th percentiles on each normalized volume, increasing organoid contrast relative to the background. Previous studies have shown that speckle noise in OCT scans has a Poisson distribution [25]. To improve the effectiveness of the subsequent filtering steps, the noise is redistributed from Poisson to Gaussian via square root transformation:

(3) $$V^{\prime \prime }\;=\;\sqrt{V^{\prime }}$$

where $V^{\prime }$ is the intensity-normalized and stretched image data, and $V^{\prime \prime }$ is the transformed data. This transformation is ultimately inverted by squaring the image volume after the last preprocessing step. The effect of the square root transformation on noise intensity distribution is illustrated in Figure 3. 

Subsequently, we perform the sharpening of the volumes by subtracting a smoothed version (U), called “un-sharpening mask” [26], to highlight the edges of the organoids:

(4) $$V_{u}\left(x,y,z\right)=\frac{b}{2b-1}V^{\prime }\left(x,y,z\right)-\frac{1-b}{2b-1}U\left(x,y,z\right)$$

where the parameter b defines the relative weight between the original volume and its filtered version. In this work, we set b equal to 23. U is the filtered version of the volume obtained by applying the Gaussian filter $g\left(x,y,z,\sigma \right)$ with parameter σ set equal to 3:

(5) $$g\left(x,y,z,\sigma \right)=\frac{1}{2\pi \sigma^{2}}\exp\left(-\frac{x^{2}+y^{2}+z^{2}}{2\sigma^{2}}\right)$$

**Figure 4:**
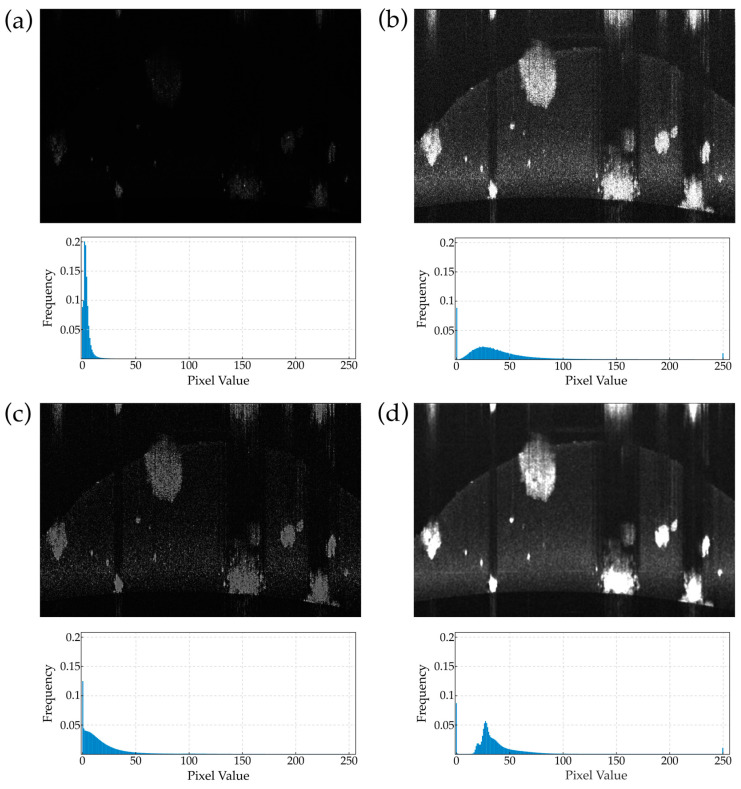
The preprocessing steps of the algorithm: (**a**) Original OCT image. (**b**) Image after histogram normalization and contrast stretching to enhance clarity. (**c**) Image following the application of a sharpening filter to accentuate specific features. (**d**) Resultant image after median filtering to reduce noise. Below each panel, the luminance histogram illustrates the distribution of intensity values, highlighting the effects of each preprocessing step.

Subsequently, we apply a median noise reduction filter with a cubic kernel of size 7. Finally, we convert the volumes to uint8 format. Figure 4 displays the main preprocessing steps of the algorithm. 

### 2.3. Deep Learning Segmentation and Postprocessing

**Figure 5:**
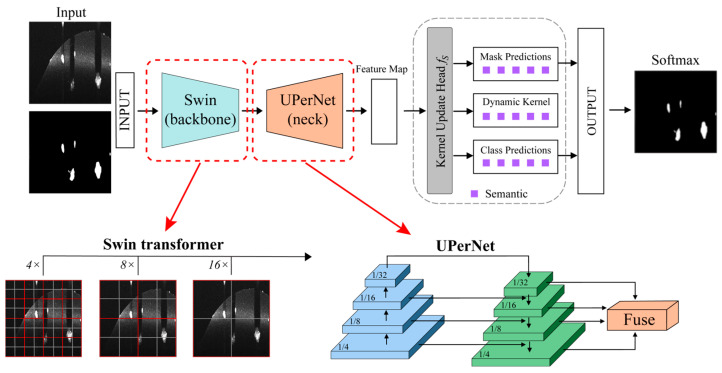
An overview of the employed K-Net architecture for organoid segmentation.

The network architecture used for organoid segmentation is a K-Net [27] implemented using the MM Segmentation framework [28]. K-Net is an encoder–decoder network with a SwinTransformer [29] backbone and a kernel generation decoder head. The architecture of the employed model is shown in Figure 5. Since the datasets consist of 3D OCT volumes, single 2D slices were extracted from each volume for network training. This allows for the use of a 2D architecture to handle the slice-by-slice prediction task efficiently. The Swin Transformer backbone extracts multiscale features from each input and is employed thanks to its efficiency and capability to capture long-range dependencies in 2D images. Moreover, its hierarchical structure facilitates a potential future scaling of the model to different image resolutions and complexities (e.g., fine-tuning for other types of biological organoids), and studies have shown good generalization and robustness in numerous image processing tasks [29,30]. 

The decoder head consists of a kernel generation module that leverages self-attention to capture global organoid shape contexts. It generates convolutional kernels conditioned on the input feature maps. These dynamic kernels are then used to decode semantic features [27]. Three sequential kernel update blocks further refine the features with skip connections from low-level encoder outputs. Each block updates the kernels based on preceding features. An auxiliary head is also used to complement the main prediction tower during training. It applies the same decoder but operates at 1/4 input resolution.

The choice of training data from specific timepoints during organoid development is crucial for optimizing the deep learning model’s performance. Data from days 1 and 3 were not included due to the small size of the organoids during these initial stages of growth, which poses challenges for manual segmentation tasks. The limited resolution and contrast of small organoids can hinder the accurate manual delineation of organoid boundaries. By focusing on the later stages of organoid development (day 5 onwards), where the organoids have grown to a sufficient size and exhibit more distinct morphological features, we ensure that the training data are of high quality and can effectively guide the learning process of the deep learning model. Our data preprocessing resizes all input images to 1024 × 1024. It also applies photometric distortions, flipping, and normalization for data augmentation during training. The network is trained end-to-end using a Dice loss for 100 epochs with a batch size of 4. Pretrained weights are used for initialization, and training is fine-tuned for our dataset.

During inference, the trained network is applied to each 2D slice extracted from the 3D OCT volumes. Predictions are made on a per-slice basis. As a postprocessing step, each detected organoid from all slices is further processed. First, the organoid’s volume is approximated as a sphere. Then, the spherical volume is compared to a reference volume of a sphere with a diameter of 25 μm. Any objects having a volume smaller than this reference spherical volume are removed [21].

### 2.4. Tracking Algorithm

Following the segmentation and postprocessing steps, which delineate individual organoids within the 3D volumes, our tracking algorithm is deployed to meticulously document the dynamic evolution of organoids across the entire time series. This includes capturing both growth patterns and morphological changes. 

**Figure 6:**
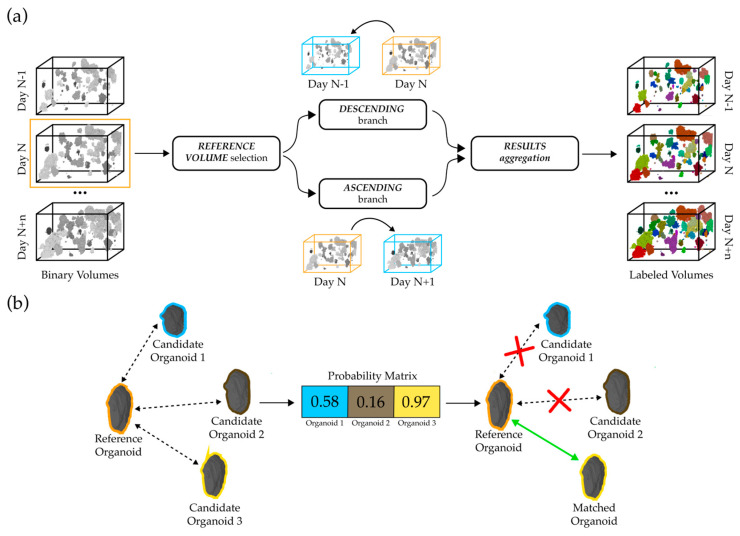
Visualization of tracking algorithm and matching process: (**a**) Overview of tracking algorithm: initial selection of reference volume, division into descending and ascending branches for forward and reverse temporal tracking, respectively, and integration of results to obtain labeled organoid volumes. (**b**) Mechanism of organoid matching: comparison of reference organoid with potential candidates, calculation of probability matrix, and determination of most probable match based on probability matrix.

At the outset, our dual branch tracking algorithm selects a reference volume from the time series, establishing an anchor point that bifurcates the tracking into ascending and descending branches, as shown in Figure 6. The selection of the reference volume is a critical step, as it determines the efficacy and accuracy of the tracking process. The reference volume is not arbitrarily fixed but is systematically optimized through experimental evaluations. Various volumes within the time series are assessed to identify which one offers the best trade-off between the number of organoids successfully tracked and the accuracy of the tracking. This optimization is crucial as it ensures a stable and consistent baseline for tracking. The final selection is based on achieving the maximum possible tracking continuity while maintaining high accuracy, assessed through a detailed three-dimensional visual analysis of the tracked organoids.

The ascending branch advances forward in time from the reference volume, tracking organoid progression to subsequent volumes, with each volume analyzed becoming the new reference for the next stage. Conversely, the descending branch conducts a reverse analysis, meticulously charting organoid growth back to the initial stages. 

During the matching phase, each organoid’s key attributes—centroid position, volume, and shape descriptors—are evaluated within the reference and subsequent evaluation volumes. Organoids in the reference volume are then compared to those in the evaluation volume based on these properties, incorporating spatial positioning, volume, and morphological characteristics, alongside the Intersection over Union (IoU) score. This comparison is supported by specific cut-off criteria to ensure the accuracy of matches. If the centroid distance between matching candidates exceeds a predefined limit, set in the range of 20 to 40 μm, that pairing is immediately excluded to dismiss potential mismatches. Additionally, the algorithm adjusts for volume oscillations between sequential scans: in the ascending branch, a decrease inside the tolerance interval is permissible to account for segmentation errors, while any increase is expected; conversely, in the descending branch, an increase inside the tolerance interval is allowed, and a general decrease in volume is expected. The tolerance interval is set in the range from 5% to 15%. Matches that fall outside these parameters for centroid distance or volume changes are assigned a zero-probability score, ensuring that only plausible matches are considered. These defined thresholds ensure that the tracking remains robust and specific, aligned with the expected biological behavior of the organoids and the precision of the segmentation process.

This comparison results in a probability score for each match, indicating the likelihood of organoid identity continuity across timepoints. The probability score P is calculated as follows:

(6) $$P\left(match\right)=\frac{1}{1+e^{-S}}$$

where S represents a composite score calculated from the weighted sum of normalized differences between key organoid attributes (centroid position, volume) and similarity metrics (shape descriptors, Intersection over Union (IoU) score). Specifically, S is defined as follows:

(7) $$S=w_{c}\cdot D_{c}+w_{v}\cdot D_{v}+w_{p}\cdot D_{p}+w_{IoU}\cdot IoU$$

where $D_{c}$, $D_{v}$, and $D_{p}$ denote the normalized differences in centroid positions, volumes, and other organoid properties, respectively. The weights $w_{c}$, $w_{v}$, and $w_{IoU}$ can range from 0 to 1, reflecting the relative importance assigned to each metric in the tracking process. A weight of 0 indicates that the attribute does not influence the matching decision, while a weight of 1 gives it full influence. Matches with high degrees of similarity are deemed high probability, reflecting a strong likelihood of matching organoid entities across volumes. It is important to notice that only matches with probability scores above a certain threshold are considered to ensure the robustness and specificity of the algorithm. 

Each probability score is then inserted in a comprehensive probability matrix, which quantitatively assesses the likelihood of matches based on the evaluated organoid properties. The process is inherently iterative, allowing for the refinement of tracking as high-probability matches are identified and prioritized. To enhance the analysis’s specificity and accuracy, organoids that have been matched are subsequently excluded from further consideration, eliminating redundancy. Furthermore, each organoid is assigned a unique numerical label within the time series, enabling the detailed analysis of individual organoid evolution over time.

Tracking organoids consistently over time posed challenges due to varying growth rates, morphological differences between organoids, and misalignment issues across timepoints. To address these challenges, the tracking algorithm parameters were optimized through a heuristic process aimed at balancing two goals: maximizing the number of organoids tracked long-term and ensuring accurate tracking throughout the dataset. For each sample dataset, numerous configurations were tested by altering the following parameters: reference timepoint, centroid distance thresholds, and volume oscillation thresholds. The configurations that resulted in the most organoids being successfully tracked over time were selected as optimal. In addition, the qualitative visual analysis of the labeled 3D renderings of the tracked organoids complemented the quantitative tracking metrics during configuration selection. This optimization process enabled the customization of the tracking algorithm to handle inherent variability in organoid growth patterns and morphology across different samples.

### 2.5. Performance Metrics

To ensure a comprehensive assessment of our proposed deep learning-based pipeline for organoid segmentation and tracking, we employed a set of widely used performance metrics, including DICE score, accuracy, sensitivity, and precision. The DICE score, which ranges from 0 to 1, provides a balanced measure of segmentation accuracy by considering both precision and recall. A higher DICE score indicates better segmentation performance. Accuracy, on the other hand, measures the overall correctness of the segmentation. Sensitivity and precision are complementary metrics that assess the model’s ability to correctly identify organoid pixels and background pixels, respectively. High sensitivity indicates that the model effectively captures most of the organoid regions, while high precision suggests that the model has a low rate of false positive predictions. These metrics were carefully selected based on their ability to accurately capture various aspects of segmentation quality and their widespread use in the field of medical image segmentation.

### 2.6. Computational Resources

The deep learning model used in this study was trained and evaluated using a high-performance computing system equipped with an NVIDIA RTX3090 with 24 GB of memory. The training process was performed using the PyTorch deep learning framework (version 1.9.0) and the Python programming language (version 3.8.5). The training of the K-Net model with a Swin Transformer backbone required approximately 6 h to complete, utilizing a batch size of 4 and running for 100 epochs. During inference, the trained model processed a single OCT volume (698 slices with dimensions of 1200 × 800 pixels) in about 30 s, enabling the efficient segmentation and tracking of organoids in real time. The inference process was performed using the same NVIDIA RTX3090 GPU, ensuring consistent performance and speed.

It is important to note that while the training process requires significant computational resources and time, the inference process is relatively fast and can be performed on less powerful hardware, such as a standard desktop computer with a modern GPU. This makes the proposed deep learning-based pipeline accessible and practical for researchers and clinicians working with OCT images of organoids.

## 3. Results

### 3.1. Segmentation Results

To select the most suitable deep learning model for our segmentation and tracking tasks, we conducted a comprehensive evaluation of several state-of-the-art architectures. We trained and compared the performance of four models: DeepLabV3+ [31], ConvNeXt [32], K-Net [27], and Swin Transformer [29]. Our choice of the K-Net architecture with a Swin Transformer backbone was based on its superior performance in capturing long-range dependencies and generating dynamic convolutional kernels conditioned on the input feature maps. In Table 2, the segmentation performance metrics for the test set are detailed, demonstrating the algorithm’s effectiveness across various measures. The DICE score is reported at 0.726 ± 0.152, highlighting the algorithm’s accuracy in segmentation tasks. Additionally, the reported accuracy of 0.996 ± 0.002 underscores the high overall correctness of the segmentation, while sensitivity and precision are noted at 0.740 ± 0.202 and 0.743 ± 0.132, respectively, indicating the algorithm’s ability to correctly identify organoid pixels and differentiate them from the background.

**Figure 7:**
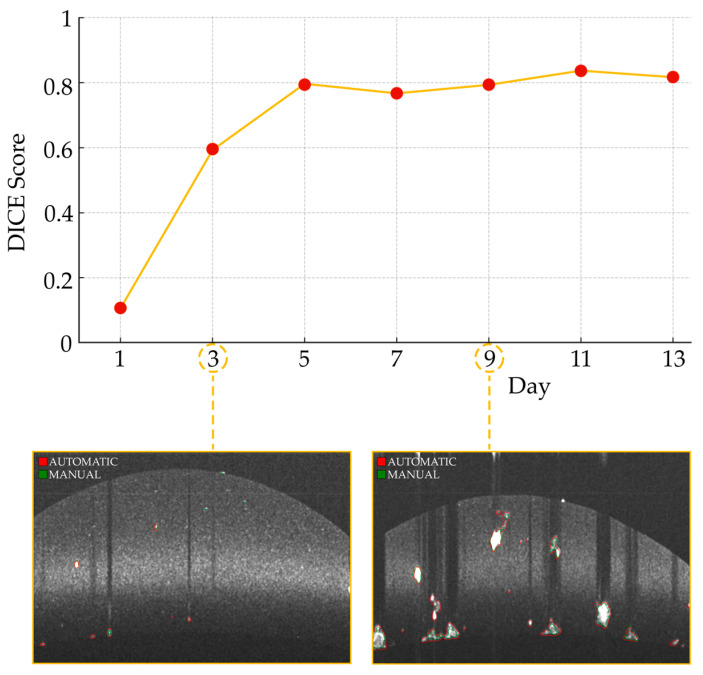
Daily DICE score progression in the time series with segmented slice examples. The trend line illustrates the variation in the DICE score across the days, reflecting the performance of the organoid segmentation algorithm at each timepoint. The insets highlight slices from days 3 and 9, with automatic segmentation contours marked in red and manual ground truth contours in green.

Figure 7 provides a visual representation of the segmentation process across different developmental stages of the organoids, with example slices from days 3 and 9. This illustration not only showcases the algorithm’s capability to adapt as the organoids grow but also emphasizes the consistent performance from day 5 onwards, when the organoids become more distinguishable from the surrounding noise. It is noteworthy that the lower performance observed during the early days is attributed to the small size of the organoids and their resemblance to surrounding noise, which poses a challenge for segmentation accuracy. This factor contributes to the initial variability in performance metrics, as the algorithm gradually improves its capability to differentiate between organoids and noise with the growth of the organoids.

### 3.2. Tracking Results

**Figure 8:**
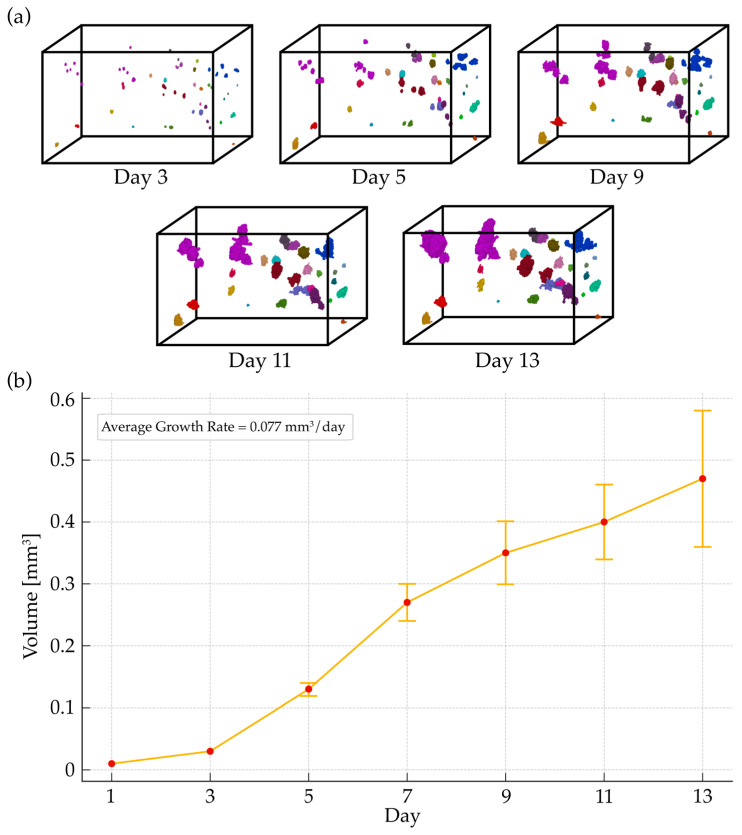
Tracking results and growth trend analysis: (**a**) Tracked volume sequence: labeled volumes from day 3 to day 13, illustrating organoid growth. Each frame shows organoids’ development, highlighting the tracking algorithm’s efficacy in capturing growth patterns. (**b**) Growth trend graph: quantifies the organoids’ volumetric expansion over time, presenting the mean organoid volume per day, with error bars for standard error and a growth rate of 0.077 mm^3^/day.

After the segmentation phase and the generation of automatic masks, the tracking algorithm was employed to elucidate both the collective and individual dynamics of organoids within the test set sample w3. Figure 8a offers a detailed visual account of organoid development across selected timepoints, providing labeled volumes that map the growth trend of the organoids. This sequence visually demonstrates an evident increase in both the size and complexity of the organoids, highlighting the algorithm’s proficiency in adapting to their evolving morphological characteristics. In finding the optimal configuration for the tracking algorithm, a tuning process was undertaken, varying all parameters such as centroid distance threshold, volume oscillation tolerance, weights for matching criteria, and the reference day. The configuration that achieved the best results included a centroid distance threshold of 30 μm and a volume change tolerance of 10%. The optimized weights were equal to 0.9 for the centroid position, 0.6 for volume changes, and 1 for the Intersection over Union (IoU) score. These adjustments specifically addressed the challenge of misregistration across different imaging days. For the test set (i.e., sample w3), which includes days 1, 3, 5, 7, 9, 11, and 13, the tuning process revealed that day 3 was the most effective starting point for tracking. This outcome was primarily due to the lower incidence of fusion events on day 4, which could complicate tracking in later days by causing a significant loss of identifiable organoids. Opting for an earlier reference day thus allowed for a more stable and clearer baseline for the tracking process. The optimized configuration enabled the algorithm to successfully track a total of 142 organoids across the entire time series when an initial 253 organoids were located in the reference volume (56%). In parallel, Figure 8b presents a quantitative analysis of the organoids’ mean volume over time, incorporating error bars to denote the standard error and capture the variability of these measurements. The graph reveals a clear growth trend, characterized by an average growth rate of 0.077 mm^3^/day. This rate quantifies the volumetric expansion of the organoids throughout the observation period, serving as a pivotal metric for quantitatively assessing organoid development. It attests to the tracking algorithm’s capability in accurately quantifying growth dynamics.

## 4. Discussion

OCT imaging is emerging as a highly promising technique for organoid analysis, offering important advantages over other imaging modalities thanks to its non-destructive, label-free, and high-resolution imaging capabilities. The joining of OCT imaging and organoid analysis will be crucial in the future for precision medicine and drug development, by enabling researchers to monitor the growth, development, and function of individual organoids with unprecedented detail [33].

In this study, an algorithm was proposed for segmenting and tracking the growth of cancer organoids in OCT volumes. The steps include data acquisition, preprocessing through image normalization, training a K-Net CNN for the segmentation of single organoids at multiple timepoints, and finally tracking single organoid growth over time. The original OCT volumes typically show poor contrast, unclear boundaries, and a noticeable amount of noise. To mitigate these issues, the proposed preprocessing stage was included to improve the visible quality of the volumes and to increase the contrast between the background and the organoid structure. For the segmentation phase, a state-of-the-art segmentation model was implemented, based on the K-Net. The Swin Transformer backbone within the architecture is fundamental as it aids in extracting multiscale features from each input image and in capturing long-range dependencies in 2D images [34,35]. The accurate segmentation of single organoids obtained by the K-Net then served as a stepping-stone for the crucial task of tracking single organoids at multiple timepoints over 13 days.

The accuracy of organoid segmentation is influenced by factors such as size, shape, and internal structure [21]. Hence, it is important to mention that the training and validation of the segmentation network were conducted on samples acquired from day 5 to day 13 after the initial organoid cultivation. On the other hand, the test set included multiple days that were not present in the training and validation set, specifically in the early days right after the initial cultivation. This provides an intrinsic hurdle to overcome for the segmentation network, yet the performance results for the early days are in line with previous studies that have shown that smaller organoids are more challenging to segment accurately due to their small size and similarities to background noise [21]. The segmentation performances obtained on the days that were also included in the samples for the training and validation sets were, as expected, satisfactory (DICE = 0.80).

A crucial aspect of OCT imaging and organoid analysis is the ability to longitudinally track organoid growth. The complexity of the tracking process is compounded by the fact that different wells exhibit slightly different morphological characteristics, and some organoids demonstrate unique biological behaviors, such as varying growth rates, which can complicate the tracking process. To address these challenges and reduce cases of mis-tracking, the proposed solution takes into consideration various key organoid attributes such as the centroid position, volume, and shape descriptors. This proved to be key in accurately matching organoids at different timepoints and allowed us to deal with challenging merging and division events that may inevitably occur. Figure 8a shows the single tracked organoids with a unique color code. The average growth rate of 0.077 mm^3^/day is both quantitatively and qualitatively appreciated, along with the fact that not all organoids present the same growth pattern. Some organoids (e.g., the purple-coded organoid) in Figure 8a shows a rapid growth rate, whereas others show a much smaller growth rate, especially on later days (e.g., the teal-coded organoid). The extensive tuning of algorithm hyperparameters, informed by quantitative and 3D visual assessments, optimized the tracking. This calibration allows the algorithm to adapt to diverse organoid conditions, maintaining good robustness. Studies have provided evidence that tumor organoids’ response to therapy are similar to that of the patients they were derived from, reflecting the genetic and acquired mutational make-up [36,37]. However, these retrospective studies were only true for a subset of patients. This may arise due to analyzing the bulk organoid culture via gold standard 2D imaging or ATP-based endpoint viability assays, thereby not taking the heterogeneity of cancer organoids within a culture into account [38]. Allowing for the assessment of single organoid growth kinetics—in contrast to bulk culture—upon treatment is crucial to improve the analysis of drug sensitivity and tackle the intratumoral heterogeneity of therapy response [8]. In this work, we lay the foundation for an AI-assisted segmentation and analysis pipeline to track the behavior of individual cancer organoid structures. This will have a high impact on the identification of rare, rapidly growing organoids that so far have remained hidden in bulk analysis approaches and will open new avenues for the early clonal identification of rare drug-resistant tumor cells, which may provide the seed of therapy resistance observed in patients.

The proposed deep learning-based pipeline for organoid segmentation and tracking provides quantitative insights into the key aspects of organoid growth and development over time. Specifically, the segmentation results allow for the assessment of organoid size, volume, shape, and morphology, which are crucial indicators of growth rate, viability, and overall health. Tracking organoids across multiple timepoints facilitates a longitudinal analysis of growth rates and morphological changes. Together, segmentation and tracking allow for an automatic quantification of the dynamic processes related to organoid formation and differentiation. Thereby, such deep learning-based pipelines can heavily assist in interpreting the results obtained from cancer organoids screened against a library of drug compounds, especially when combined with gene expression analysis, protein–protein interactions, and pathway information to enhance the power of drug sensitivity prediction [39]. Generating patient-derived organoids and subjecting them to drug screens accompanied with imaging techniques such as OCT coupled with deep learning-assisted analysis could facilitate the establishment of pipelines for a faster and individual treatment regimen [8,38,40]. Using patient-derived organoid platforms as “avatars” for predicting drug response in precision medicine provides an unmatched prospect to improve preclinical drug discovery [41]. However, some hurdles still need to be tackled for use in clinical settings driving patient care, such as the lack of standardized sample isolation and automation for culture approaches which may result in experimental variations across different research labs [41,42]. Likewise, for deep-learning approaches, no unified framework exists to compare drug response models in a standardized fashion [39]. Albeit patient-derived organoids allow for individualized treatment selection, discussions on ethical constraints related to the utilization of organoids in drug testing may derive from the safeguarding of patient privacy and the high cost of development, as both of these aspects make this approach only available to a subset of patients [43].

Future work will aim to substantially increase the dataset size which will improve segmentation accuracy and robustness across timepoints. Furthermore, incorporating additional imaging modalities, such as fluorescence microscopy or histological staining, could provide valuable information about the internal structure and composition of organoids, enabling a more comprehensive understanding of organoid biology. Larger volumes of training data will enhance the networks’ capability to generalize to new samples, especially improving performance on early-stage organoids. The approach presented here can also easily be employed to assess differences between organoid cultures that are, for example, treated with a specific drug and cultures that do not receive any drug treatment. This can provide an important basis for drug sensitivity evaluation. Currently, the organoid culture was removed from an incubator shortly prior to imaging and returned afterward, which still may allow for a growth kinetics difference due to temperature change. To further tailor the system for organoid culture applications, an incubator and automatic stage could be included to enable imaging at cell culture conditions (37 °C, 5% CO_2_) at pre-specified positions and time to reduce tedious manual work. Moreover, once organoids grow bigger in size, the imaging penetration depth is limited. This can be tackled by using acoustic tweezers [44] or a rotating stage to turn the sample [23]. Another potential area for future optimization is the development of instance segmentation techniques to handle the challenges posed by organoid fusion at later stages of growth. As organoids continue to grow and expand, they may merge with neighboring organoids, making it difficult to accurately distinguish and segment individual structures. Applying instance segmentation approaches and implementing algorithms specifically designed for separating fused organoids could be an interesting avenue for future research, enabling the more precise tracking and analysis of organoid development over extended periods. Additionally, future improvements in our tracking algorithm will focus on the dynamic and automatic optimization of parameters tailored to each individual organoid, ensuring the tracking process continuously adapts to the unique conditions of each experiment. Finally, this study presents a segmentation and tracking method for organoids that have been cultured following a specific protocol and relative to a specific tissue. While the overall approach can be easily extendable to other types of organoids and experimental circumstances, the segmentation network and tracking algorithm would need to be fine-tuned to take into consideration macro-differences in organoid morphology, size, and organoid density within the analyzed culture.

In addition to segmentation and tracking, future work will also focus on developing radiomic applications for analyzing the morphology, heterogeneity, and texture of organoids in OCT volumes. Radiomics involves extracting quantitative features from medical images and has shown promise in determining tumor status or response to treatment in cancer research [45]. Applying these techniques to organoids could provide valuable insights into their developmental trajectories and overall health status. This information would be crucial for personalized medicine applications, where understanding the unique characteristics of tissues is essential for designing effective treatments.

## 5. Conclusions

In conclusion, OCT imaging and organoid analysis are emerging techniques that will play a critical role in precision medicine. Advanced imaging techniques such as deep learning-based segmentation have shown promise in providing accurate segmentation that can then be employed to track the growth and development of single organoids over time. The framework presented provides an important tool for longitudinal organoid analysis, which will help enable applications in drug screening and precision medicine. Specifically, the approach can be used to evaluate drug effects by comparing segmentation and tracking metrics between treated and untreated organoid cultures. Future work should focus on developing applications to further enhance our understanding of these complex structures’ biology and function, ultimately leading to more effective personalized treatments.

## Figures and Tables

**Table 1:** An overview of the datasets employed.

Subset	Sample ID	Days (Each Day Represents One Volume)	Dimension	Total Number of Images
Training	w1	d5, d7, d11, d13	1200 × 800 × 698	5584
	w2	d5, d7, d9, d11	1200 × 800 × 698	
Validation	w1	d9	1200 × 800 × 698	698
	w2	d13	1200 × 800 × 698	5584
Testing	w3	d1, d3, d5, d7, d9, d11, d13	1200 × 800 × 698	

**Table 2:** Performance metrics on test subset.

Method	DICE Score	Accuracy	Sensitivity	Precision
DeepLabV3+ [31]	0.702 ± 0.159	0.995 ± 0.004	0.786 ± 0.223	0.682 ± 0.145
ConvNext [32]	0.700 ± 0.170	0.994 ± 0.003	0.785 ± 0.232	0.684 ± 0.148
K-Net [27]	0.702 ± 0.149	0.995 ± 0.003	0.786 ± 0.209	0.673 ± 0.146
Swin [29]	0.698 ± 0.184	0.994 ± 0.003	0.775 ± 0.244	0.678 ± 0.163
K-Net+Swin (proposed)	0.726 ± 0.152	0.996 ± 0.002	0.740 ± 0.202	0.743 ± 0.132

## Data Availability

The data presented in this study are available upon valid request.
